# Safety and efficacy of fenproporex for obesity treatment: a systematic review

**DOI:** 10.1590/S1518-8787.2016050006208

**Published:** 2016-05-06

**Authors:** Francisco José Roma Paumgartten, Sabrina Schaaf Teixeira Costa Pereira, Ana Cecilia Amado Xavier de Oliveira

**Affiliations:** Departamento de Ciências Biológicas. Escola Nacional de Saúde Pública. Fundação Oswaldo Cruz. Rio de Janeiro, RJ, Brasil

**Keywords:** Anti-Obesity Agents, adverse effects, Appetite Depressants, toxicity, Amphetamine, contraindications, Drug Utilization, Off-Label Use, Effectiveness, Safety, Controlled Clinical Trial, Review

## Abstract

**OBJECTIVE:**

To evaluate clinical evidence on the safety and efficacy of fenproporex for treating obesity.

**METHODS:**

MEDLINE, LILACS and Cochrane Controlled Trials Register were searched as well as references cited by articles and relevant documents. Two authors independently assessed the studies for inclusion and regarding risk of bias, collected data, and accuracy. Eligible studies were all those placebo-controlled that provided data on the efficacy and safety of Fenproporex to treat obesity.

**RESULTS:**

Only four controlled studies met the inclusion criteria. One randomized, placebo-controlled trial on Fenproporex was found on electronic databases. Three placebo-controlled studies (in non-indexed journals) were identified by hand-searching. Patients with cardiovascular and other comorbidities were excluded in all studies. Trials lasted from 40 to 364 days and doses ranged from 20 to 33.6 mg/d. All controlled studies found that weight loss among Fenproporex-treated patients was greater than that produced by the placebo, but drug effect was modest. Fenproporex produced additional weight reductions of 4.7 kg (one year), 3.8 kg (six months) and 1.55 kg (two months) in average, in relation to diet and exercise only (three trials). Insomnia, irritability, and anxiety were the most frequently reported side effects in the four studies.

**CONCLUSIONS:**

There is a paucity of randomized, placebo-controlled trials on Fenproporex and those identified here present major methodological flaws. These studies suggest that Fenproporex is modestly effective in promoting weight loss. Nonetheless, they failed to provide evidence that it reduces obesity-associated morbidity and mortality. Data from these studies are insufficient to determine the risk-benefit profile of Fenproporex. Abuse potential and amphetamine-like adverse effects are causes for concern.

## RESUMO

**OBJETIVO:**

Avaliar a evidência clínica de segurança e eficácia do Fenproporex para tratamento da obesidade.

**MÉTODOS:**

Pesquisamos publicações em qualquer idioma nas bases Medline, Lilacs *Cochrane Controlled Trials Register* e também referências citadas por artigos e documentos relevantes. Dois autores avaliaram independentemente os estudos para inclusão e quanto ao risco de viés, dados coletados e precisão. Foram elegíveis estudos controlados com placebo que forneceram dados sobre a eficácia e segurança do Fenproporex para tratar a obesidade.

**RESULTADOS:**

Apenas quatro estudos controlados preencheram critérios de inclusão. Um estudo placebo-controlado aleatorizado do Fenproporex foi encontrado nas bases eletrônicas. Três estudos controlados (em periódicos não indexados) foram identificados por buscas manuais. Pacientes com comorbidades (cardiovasculares ou outras) foram excluídos em todos os estudos. A duração dos estudos foi de 40 a 364 dias, com doses de 20 a 33,6 mg/d. Todos os estudos controlados encontraram maior perda de peso entre pacientes tratados com Fenproporex, comparados aos que receberam placebo, mas o efeito foi modesto. O Fenproporex causou reduções adicionais de peso de 4,7 kg (após um ano), 3,8 kg (após seis meses) e 1,55 kg (após dois meses), em média, em relação à dieta e exercício apenas (três ensaios). Insônia, irritabilidade e ansiedade foram os eventos colaterais mais frequentes nos quatro estudos.

**CONCLUSÕES:**

Ensaios clínicos placebo-controlado aleatorizado do Fenproporex são escassos e os estudos controlados identificados apresentam importantes falhas metodológicas. Esses estudos sugerem que o Fenproporex é modestamente eficaz em promover perda de peso. Entretanto, eles não fornecem evidências de que o Fenproporex atenua a morbidade e mortalidade associada à obesidade. Esses estudos são insuficientes para avaliar o perfil risco-benefício do Fenproporex. Potencial de abuso e efeitos adversos do tipo anfetamínico são motivos de preocupação.

## INTRODUCTION

Fenproporex (*3-[(2-methyl-1-phenylpropan-2-yl)amino]propanenitrile*), a compound structurally similar to amphetamine, was developed as an anorectic drug in the 1960s. Notwithstanding the structural resemblance, it was claimed that fenproporex (FEN) would be devoid of amphetamines’ stimulatory effects and thus it was recommended as the anorectic drug of choice for obese patients with cardiovascular comorbidities^13^ ^,^ ^14^ ^,^ ^23^ ^,^ ^26^ ^,^ ^31^. However, Tognoni et al.^29^ and Beckett et al.^2^ found amphetamine in the brain of rats and in the urine of human volunteers treated with FEN. The observation that FEN is to a great extent (33.0% to 66.0%) converted into amphetamine in the body was confirmed by further studies^4^ ^,^ ^5^ ^,^ ^9^ ^,^ ^11^ ^,^ ^17^.

Drugs with chemical structures similar to amphetamine (i.e., amphetamine-like anorectics) are among the earliest pharmacological agents used for weight loss. In the 1930s, amphetamines were already used to treat obesity and, by the 1940s and 1950s, amphetamines alone or combined with other pharmacologically active substances (e.g., diuretics, thyroid hormones, laxatives, tranquilizers) in diet pills (also known as “rainbow diet pills” in the US) became popular therapies for overweight and obesity^7^. Since then, amphetamines have been associated with drug abuse, mood changes, psychiatric disorders, life-threatening side effects, and sudden deaths, and their use for weight loss is considered unsafe.

Despite the health concerns, the US Food and Drug Administration (USFDA) approved desoxyephedrine in mid-1940s, and phentermine, diethylpropion (or amfepramone) and benzphetamine in 1959 and 1960 as adjunct medications to treat obesity. Owing to an unfavorable risk-benefit ratio and concerns regarding abuse liability, the USFDA has never approved FEN for obesity or any other clinical indication. Although phentermine and diethylpropion remained on the market, the USFDA restricted their clinical indications to short-term (a few weeks) use as adjuncts in the management of obesity^7^ ^,^ ^8^.

In 1996, marketing authorizations of FEN and other amphetamines were harmonized within the European Community. Then, treatment with these appetite suppressants was stipulated not to exceed three months owing to risks associated with their prolonged use^a^. In the early 2000s, the European Agency for the Evaluation of Medicinal Products ordered the withdrawal of marketing authorizations for FEN, amfepramone, mazindol, and other amphetamines^24^ ^,^ ^33^. The European agency based its decision on the lack of efficacy of amphetamine-like anorectics according to the new scientific criterion for long-term efficacy of antiobesity drugs^33^. The drug manufacturers appealed to the European Court of First Instance, which annulled the agency’s ban on these amphetamine-like anorectics^24^ ^,^ ^33^. According to the European Court, “A mere change in the consensus on the efficacy of those drugs in the treatment of obesity, which is not based on any new data, does not justify withdrawal of marketing authorization”^33^. In the first decade of this century, Brazil had become one of the world’s top consumers of amphetamines and anorectic drugs. In 2011 the Brazilian National Sanitary Surveillance Agency (ANVISA) banned FEN, diethylpropion and mazindol and introduced stricter control rules for prescribing and dispensing sibutramine^b^. Two years later, however, the ANVISA regulation that prohibited amphetamine-like anorectics was annulled by a legislative decree^c^. Although the Brazilian Congress has lifted the ban on amphetamine anorectics, marketing authorizations of the pharmaceutical products containing FEN, diethylpropion, and mazindol remained cancelled (the agency edits cancelling them were not annulled by the legislative decree). Therefore, manufacturers have to submit a new drug application if they want to place these products on pharmacy shelves again.

Brazilian doctors and their associations are divided over whether amphetamine anorectics should be banned or put on stricter control rules for dispensing. They are far from being recognized as gold standard medications. However, some doctors of medical specialties dealing with obesity treatment argue that these drugs might be useful for a subgroup of obese patients who proved to be unresponsive to any other therapeutic approach^d^. The withdrawal of amphetamines from the Brazilian pharmaceutical market, however, is supported by many physicians who defend evidence-based therapeutic interventions and by most public health scientists.

This systematic review aims to provide physicians and regulators with an updated analysis of the best clinical evidence available from placebo-controlled clinical trials on the safety and efficacy of fenproporex in obesity treatment.

## METHODS

A systematic search in MEDLINE, LILACS, and the Cochrane Controlled Trials Register (CCTR) databases was conducted using the search string fenproporex (OR femproporex). The search covered the time period from the inception of each electronic database until February 4, 2015. To identify studies of interest for this review that were published in non-indexed journals, the authors also examined reference lists of original and review articles as well as reports and therapeutic guidelines issued by medical associations and regulatory agencies. To be accepted for analysis, a clinical trial had to be a placebo-controlled study on FEN safety and efficacy in the management of overweight or obesity (BMI > 30 kg/m^2^) of adolescent or adult, male or female patients. No restriction regarding duration of the trial was imposed.

Full-text articles were retrieved for all abstracts of potentially relevant studies. There was no restriction regarding the language of the article. Two researchers separately screened the titles and abstracts for inclusion and exclusion and independently reviewed the articles selected for integral reading and analysis. Examples of publications that did not meet inclusion criteria are those reporting analytical methods, animal studies, observational or retrospective human studies, case reports, uncontrolled clinical trials, case series, clinical pharmacokinetics, and narrative reviews.

Two reviewers independently extracted the data and assessed the risk of bias for each study using criteria outlined in the Cochrane Handbook for Systematic Reviews of Interventions^e^. Data extraction included details of methods, participants, eligibility, interventions, outcomes, results, and conclusions. Disagreements were resolved by discussion. To assess study quality, reviewers analyzed the following domains: generation and concealment of randomization sequence (selection bias), blinding of participants and personnel (performance bias), blinding of outcome assessors (measurement bias), and incomplete outcome data (follow-up bias). Each of the foregoing aspects of study design was categorized into low, high, or unclear risk of bias. The higher the risk of bias, the lower the methodological quality of the study. The studies classified as of unclear bias risk are those for which too few details are available to make a judgment of high or low risk.

## RESULTS

**Figure 1:**
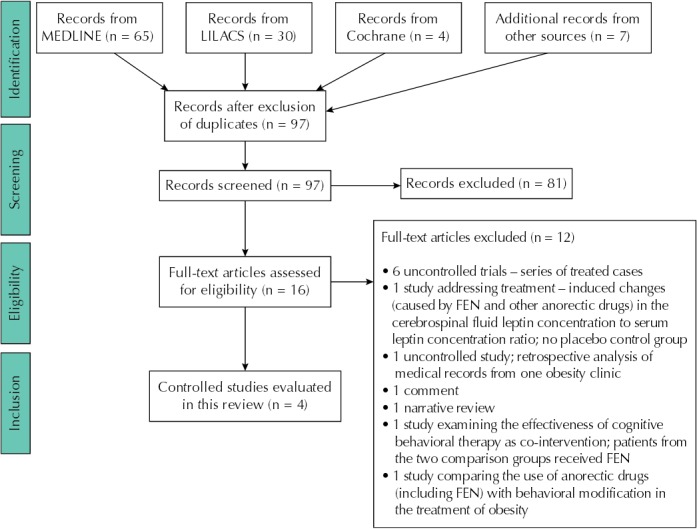
Results of search for relevant clinical studies on the efficacy and safety of fenproporex for weight loss.

The search found 65 publications in MEDLINE, 30 in LILACS, and four in the CCTR database. After excluding nine duplicates, we prescreened 97 database registries (titles or abstracts) and selected 18 of them for integral reading and analysis (Figure 1). The search identified a single randomized and controlled clinical trial on FEN^34^. We found the remaining three placebo-controlled trials of FEN by hand-searching (Table 1)^1^ ^,^ ^22^ ^,^ ^28^. They were conducted in Mexico (one) and Brazil (two), reported in the authors’ native languages (Spanish and Portuguese), and published in local medical journals. We failed to find any systematic review on the safety and efficacy of FEN.

**Table 1:** Characteristics of included studies.

Study	Design n/group	FEN dose per day	Adjunct therapy	Duration	Weight loss (kg)				Adverse effect most frequently reported

					Placebo		FEN		
	
					Mean	SD	Mean	SD	
Attié Jr and Medeiros-Neto^1^ (1972)	Nonrandomized, 10	33.6 mg	Diet + Physical exercise	60 days	-3.88	± 1.92	-5.43	± 2.09	Insomnia, dry mouth, irritability, anxiety, enhanced energy
Pinho et al.^22^ (1974)	Randomized 20-22	22.4 mg 25 mg 25 mg (SRF)	None	40 days	+0.147	± 0.51	-1.388 -2.517 -1.55	± 0.55 ± 0.52 ± 0.42	Dry mouth, anxiety, depression, gastralgia, itching, weakness, sialorrhea, precordial pain, nausea
Zaragoza et al.^34^ (2005)	Randomized, 30	20 mg (SRF) 20 mg + diazepam 6 mg (SRF)	Diet + Physical exercise	180 days	-5.1*		-8.9* -11.6*		Constipation, insomnia
Suplicy et al.^28^ (2014)	Randomized, 29 (premenopausal women)	25 mg	Diet + Physical exercise	364 days (52 weeks)	-3.1	± 4.3*	-7.8	± 6.9*	Irritability, dry mouth, constipation, anxiety, insomnia

SRF: slow-release formulations

* Intention-to-treat analysis.

We found publications of uncontrolled clinical studies, mostly case series reporting obese patients treated with FEN, on the searched databases and reference lists; however, they failed to meet the primary inclusion criterion. Table 2 shows the clinical studies on FEN not selected for reviewing and the reasons why we excluded them.

**Table 2:** Characteristics of excluded studies.

Study	Treatment			Comorbidity	Adjunct therapy	Weight loss (kg) (period; with/without diet)	Adverse effect	Reason for exclusion

	n/sex	Dose (per day)	Duration					
Warembourg and Jaillard^32^ (1968)	40 (18 M, 22 F)	33.6-44.8 mg	4-6 w	Hypertension, coronary disease, heart failure, diabetes	Diet (some patients only)	-8 (4 w) -12 (6 w)	Nervousness (3)	Uncontrolled trial
Oury and Duché^21^ (1970)	15 (13 F, 2 M) 26 (22 F, 4 M)	-	4-8 w	-	Diet (one group only)	-6 (8 w with diet) -4.7 (8 w without diet)	-	Uncontrolled trial^a^
Coronho and Pena^10^ (1973)	62	11.2, 22.4 or 33.6 mg 22.4 or 44.8 mg (SRF)	60 d	Some patients had hypertension, mild heart disease or diabetes	Diet (some patients only)	-2.24 (30 d), -3.87 (60 d) -2.84 (30 d), -5.19 (60 d)	Insomnia (5), nervousness (3), headache (2), dry mouth (2), sleepiness, fatigue, restlessness, sadness, itching, increased appetite.	Uncontrolled trial
Luz^18^ (1974)	26 F	22.4 mg	51.5 d (SD = 16.2)	Diabetes, hypertension, heart failure, cystitis, duodenal ulcer, depression (7 patients)	Diet	-5.1	Feeling of well-being or enhanced energy (19), dry mouth (10), fatigue (7), irritability (5), headache (5), itching (4), insomnia (4), euphoria (3), anxiety (3), nausea or vomit (4), dizziness, breathlessness.	Uncontrolled trial
Dinato et al.^12^ (1975)	30	50 mg (SRF)	56 d^b^	-	Diet	-4.3 (28 d) -6.2 (56 d)	Dry mouth, bad breath, nervousness, insomnia, constipation, tachycardia, breathlessness, irritability, nausea, heartburn, euphoria.	Uncontrolled trial
Chiorboli and Scazufca^3^ (1975)	42 (9 M, 33 F)	25 or 50 mg^c^ (SRF)	60 d	Hypertension (9 patients)	Diet	-4.1 (30 d) -7.4 (60 d)	Dry mouth, (22), nervousness (18), sleeplessness (7), constipation (18), nausea (13).	Uncontrolled trial
Hertel and Fallot-Burghardt^14^ (1978)	50 F	-^d^	9 w	Hypertension, diabetes	Diet	-2.8 (6 w) -4.6 (9 w)	No report of adverse effects (35); mild adverse effects (10), severe adverse effects leading to treatment discontinuation (5).	Uncontrolled trial
Rodrigues et al.^25^ (2002)	10 F	25 mg	60 d	-	Diet	-7.6 (60 d) Approx. -7.0% of the initial BW	-	Effect of treatment on CSF/serum leptin ratio; no placebo control group
Machado et al.^19^ (2002)	40 F	25 or 50 mg (SRF)	6 mo	None	Diet + PE (+ BT)^e^	-13.1 or -15.5% of the initial BW -15.7 or -15.7% of the initial BW (+ BT)	Dry mouth, insomnia, irritability, headache, palpitations.	Study tested the effect of adjunct BT; no placebo control group
Horie et al.^15^ (2010)	44 F 7 M^f^	-	6-24 mo	-	-	-	One episode of atrial flutter was diagnosed in a patient taking FEN.	No placebo control group; retrospective analysis of medical charts

M: males; F: females; SRF: slow-release formulation; w: weeks; d: days; mo: months; PE: physical exercise; BT: cognitive behavioral therapy; BW: body weight; CSF: cerebrospinal fluid

^a^ The trial was considered “uncontrolled” because only 3 F patients (the “control” group) were treated with placebo.

^b^ Plus 28 days on diet after drug treatment discontinuation.

^c^ 50 mg for the most obese patients.

^d^ One tablet 2-3 times a day, amount of FEN per tablet not informed.

^e^ Both groups were treated with diet + PE + FEN, one group additionally received BT.

^f^ Patients received different weight loss medicines, including FEN.

- : Not informed or not applicable

Table 2 also summarizes the details on the design and results of some case series. All these uncontrolled trials concluded that FEN was effective in promoting weight loss (in comparison with patients’ pretreatment body weight). Nonetheless, since participants were also encouraged to stick to a low-calorie diet, data from case series did not display the extent to which the adjunct therapy and other uncontrolled factors contributed to treatment-associated weight reduction.

In Attié Jr and Medeiros-Neto’s nonrandomized short-term (two-month) trial^1^, healthy obese patients (85.5% women) were arbitrarily assigned to placebo (n = 17) or FEN (33.6 mg/d; n = 13)^f^ groups. The researchers encouraged all patients to start a low-calorie diet (1,000 calories/day) and clinically evaluated them after 30 and 60 days of treatment. Due to dropouts before the first scheduled examination (by the end of the fourth week of treatment), conclusions were based on an analysis of 10 patients per group. After two months, mean weight loss observed among FEN-treated patients (-5.43 kg; SD = 2.09) was greater than that recorded among placebo-treated patients (-3.88 kg; SD = 1.92). A reduction of serum lipoprotein and total lipid levels was noted in the FEN-treated group but not in the placebo-treated group. Glucose and cholesterol serum levels, however, remained unaltered in both groups. The side effects most frequently reported by patients of the FEN-treated group were insomnia (30.0% *versus* 10.0% in the placebo group), dry mouth (60.0% *versus* 20.0%), irritability (50.0% *versus* 20.0%) and anxiety (40.0% *versus* 10.0%).

The randomized short-term (40-day) trial conducted by Pinho et al.^22^ was a double-blind controlled trial evaluating three distinct formulations of FEN (one simple and two slow-release formulations). Exclusion criteria were comorbidities (heart, liver, hematologic or psychiatric diseases, or peptic ulcers), pregnancy, and age under 12 years. Eighty-four obese patients (70 females, 14 males, 11-60 years old) were allocated at random to the placebo or one of the three FEN-treated groups (21 per group). Authors did not inform how they conducted the random assignment. Patients were asked not to change their dietary habits during the 40-day treatment period. Four patients of the placebo group and 15 of the FEN-treated groups did not attend the scheduled examinations, resulting in the following numbers of evaluated patients: placebo = 17; FEN = 16 (22.4 mg/d, simple), 18 (25 mg/d, intermediate release rate), and 14 (25 mg/d, slow release rate). All FEN-treated groups showed a small albeit statistically significant weight reduction, while the body weight of the placebo group remained practically unchanged (Table 1). No consistent effect of FEN on postprandial blood glucose or lipid parameters was found. The most frequent adverse effects reported by patients treated with FEN were dry mouth (30.7% *versus* 9.8% in the placebo group), sialorrhea (6.2% *versus* 0.0%), itching (12.8% *versus* 7.8%), precordial chest pain (2.3% *versus* 0%), anxiety (12.0% *versus* 2.0%) and depression (10.7% *versus* 3.9%).

Zaragoza et al.’s randomized short-term (six-month) trial^34^ was a double-blind controlled study that evaluated the weight reduction promoted by FEN (20 mg/day, slow-release formulation) and by a FEN (20 mg/day) plus diazepam (6 mg/day) fixed-dose combination. Patients with hypertension, cancer, endocrine, liver or kidney diseases as well as pregnant and nursing women were excluded. Ninety obese adult patients (17 men, 73 women, 30 per group, aged 18-60 years) were assigned to one of three groups (placebo, FEN or FEN+diazepam) and treated for six months. The authors informed that the sponsor (a pharmaceutical company) carried out the randomization and no additional information was given. Pharmacological treatment was prescribed as adjunct therapy to low-calorie diet and increased physical activity (30-min walk every day). Patients were evaluated clinically every month during the treatment period and one month after the interruption of drug or placebo administration. The six-month treatment was completed by 24 (80.0%), 22 (73.0%), and 28 (93.0%) of the patients (30 per group) allocated in the placebo, FEN and FEN+diazepam groups, respectively. At day 180, mean percentages of body weight reduction (intention-to-treat) were -5.4% in the placebo group, -11.1% in the FEN group, and -12.6% in the FEN+diazepam group. Losses to follow-up were as high as 26.6%, 30.0% and 6.6% for the placebo, FEN and FEN+diazepam groups, respectively. The most frequent adverse effects reported by patients (FEN, FEN+diazepam *versus* placebo) were constipation (53.3%, 56.7% *versus* 20.0%), paleness (16.7%, 10.0% *versus* 0%), arthralgia (30.0%, 46.7% *versus* 20.0%), cramps (13.3%, 20.0% *versus* 0%), blurred vision (16.7%, 6.7% *versus* 0%), insomnia (36.7%, 26.7% *versus* 13.3%), and tinnitus (6.7%, 16.7% *versus* 3.3%). Statistical analysis did not show differences between placebo and FEN-treated patients regarding systolic and diastolic blood pressure, heart rate, and lipid profile (triglycerides, high-density lipoprotein, low-density lipoprotein, total cholesterol levels) during or after treatment.

Suplicy et al.^28^ conducted a randomized long-term (52-week) study to compare the efficacy (magnitude of weight loss) and safety of five drugs (one of which FEN) that act on the central nervous system in the management of obesity. Only obese premenopausal women (aged 18-50 years) were eligible. For the trial, the researchers excluded women with uncontrolled hypertension, diabetes, history of significant morbidity (gastrointestinal, cardiac, renal, hepatic, pulmonary, neurological, endocrine diseases or cancer, alcoholism, and drug abuse), major depression, and generalized anxiety, besides those who intended to get pregnant. The authors reported that eligible patients (180 obese women) were randomly assigned to placebo and treatment groups but did not inform the method used to generate random allocation sequences. Six patients withdrew their consent to participate after the randomized assignment; thus, intention-to-treat analysis was restricted to 174 patients who were treated over 52 weeks with daily doses of FEN 25 mg (n = 29), diethylpropion 75 mg (n = 28), mazindol 2 mg (n = 29), sibutramine 15 mg (n = 30), fluoxetine 20 mg (n = 29), or placebo only (n = 29). Diet and increased physical activity were encouraged as adjunct therapies for promoting weight loss. Only 15 of 29 (48.0%) and 23 of 31 (74.0%) patients randomly assigned to placebo and FEN-treatment groups, respectively, completed the study. After the random assignment, two women allocated in the FEN group withdrew their consent and were excluded from intention-to-treat analysis. According to the authors, dropouts occurred predominantly within the first six months of the trial and the most frequent reasons were “therapeutic failure” (placebo *versus* FEN: 6 *versus* 1), “noncompliance” (4 *versus* 2), side effects (3 *versus* 2) and pregnancy (1 *versus* 1). The mean weight loss achieved by patients treated with FEN (-7.8 kg, SD = 6.9) was greater than that achieved by patients of the placebo group (-3.1 kg, SD = 4.3). Except for the antidepressant fluoxetine, the weight-reducing effect of which (mean of -2.5 kg, SD = 4.1) did not differ from that of the placebo, all other drugs caused weight loss (diethylpropion: -10.0 kg, SD = 6.4; mazindol: -7.4 kg, SD = 4.9, sibutramine: -9.5 kg, SD = 5.9) comparable to that achieved with FEN. The proportion of patients who complained of “irritability” was clearly augmented among those treated with FEN and diethylpropion compared with those receiving placebo (33.3% and 28.6% *versus* 3.6%). Dry mouth (51.7% *versus* 28.6%), constipation (17.2% *versus* 3.6%), insomnia (24.1% *versus* 3.6%), and anxiety (20.7% *versus* 7.1%) were also reported by patients (FEN *versus* placebo). The authors informed that they failed to mask commercial pharmaceutical dosage forms and thus blinding of treatment groups was flawed in this placebo-controlled trial.

In summary, all placebo-controlled studies consistently indicated that treatment with FEN (20 to 33.6 mg/day) caused a weight loss greater than that achieved with diet and physical exercise alone. Nonetheless, the magnitude of the body weight reduction produced by FEN in addition to that caused by the placebo was modest, i.e., < 2 kg in two months, and < 5 kg in six months or one year of treatment. No clear beneficial effects of FEN on blood pressure, blood glucose, and lipid parameters were found in any study. The few controlled trials identified in this comprehensive literature search were not designed to provide direct evidence of a possible effect of FEN in preventing or reducing obesity-associated morbidity or mortality. The side effects most frequently reported by FEN-treated patients, on the other hand, were consistent with the typical (stimulatory) effects of amphetamines on the central nervous system (e.g., irritability, insomnia). Due to the small sample sizes (n = 10, 21, 29 and 30), these placebo-controlled studies were clearly underpowered to detect differences between the placebo and FEN groups regarding the incidence of side effects.

## DISCUSSION

This systematic review showed that there is a paucity of randomized, placebo-controlled trials on the efficacy and safety of FEN for treating overweight (BMI > 25 kg/m^2^) and obesity (BMI > 30 kg/m^2^). A comprehensive search in the MEDLINE, LILACS, and Cochrane Controlled Trial Register databases found a single randomized, controlled clinical trial published in 2014, while three placebo-controlled studies, one of which nonrandomized, were identified by hand-searching for studies of interest published in non-indexed medical journals. The four controlled trials consistently indicated that FEN induced body weight losses greater than that associated with placebo use. A set of methodological flaws, however, vitiates conclusions based on their findings. Randomization, blinding, and handling of missing data due to attrition are flawed in these clinical trials. Moreover, all studies are underpowered to detect differences between control and FEN-treated groups regarding the occurrence of adverse effects.

Random assignment and concealed allocation of participants are considered the best protection against systematic differences between baseline characteristics of groups being compared. Not using concealed random allocation, which prevents foreknowledge of group assignments, entails a risk of bias in allocating interventions to participants. Nonrandom group assignments made by clinical investigators can be related to prognosis and responsiveness to treatment. However, it is generally difficult to predict the extent to which a nonrandom assignment of patients to trial groups influences the magnitude and even the direction of the effect of an intervention. Since these reports of placebo-controlled trials gave insufficient information on the randomization and allocation concealment processes, it cannot be ascertained whether objectives of randomization were successfully accomplished.

Attrition rates are generally high in long-term randomized, controlled clinical trials on appetite suppressants and limit interpretation of data on their efficacy and safety. Loss to follow-up leads to incompleteness of outcome data evaluated at the end of a trial. Moreover, if one arm of the trial has an attrition rate higher than the other, the randomization is impaired. Attrition bias may occur if patients who completed the trial are systematically different from those who failed to do it. Systematic errors caused by attrition, however, can be reduced or avoided if analysis of outcome data is based on initial random assignment (i.e., intention-to-treat analysis) and not on the treatment eventually received, and investigators use imputation methods (e.g., last observation carried out forward) to handle missing data. Only two studies^28^ ^,^ ^34^ employed intention-to-treat analysis to evaluate whether FEN was more effective than placebo in promoting weight loss.

Blinding the intervention received by the participants is also crucial for avoiding biases. A flawed blinding may result in knowledge of which intervention was received and thus entails a substantial risk of differences between groups in the amount of attention and ancillary treatments (performance bias) or in how outcomes were determined (detection bias). Along this line, a Consolidated Standards of Reporting Trials (CONSORT) statement calls for explicit reporting of “how the success of blinding was evaluated”^30^. Suplicy et al.^28^ reported that they failed to adequately mask placebo and FEN-containing capsules and thus participants, physicians, and outcome assessors might have distinguished the intervention received by each patient. Pinho et al.^22^ informed that the placebo contained talc and starch but gave no additional detail on how placebo and FEN formulations were masked. Attié Jr and Medeiros-Neto^1^ and Zaragoza et al.^34^ did not describe how they masked placebo and FEN formulations. None of the four studies adequately reported the blinding status of each key trial person and one can assume that the authors did not test the success of blinding either.

Finally, a major weakness common to all these trials is the small size of placebo and FEN-treated groups for evaluating drug safety. Two of them^1^ ^,^ ^22^ failed to estimate the minimum sample size required to achieve sufficient statistical power to detect clinically relevant differences between groups. Suplicy et al.^28^ determined that at least 30 patients per group were required to compare efficacies and reported the assumptions taken to estimate this minimum group size. They estimated the minimum number of subjects per group for achieving statistical power (power = 0.8, α-error = 0.05) to detect the primary efficacy outcome (weight loss). However, studies with group sizes ≤ 30 patients (intention-to-treat) might have been underpowered to detect clinically relevant increases in the incidence of adverse effects. For instance, if an adverse effect occurs in 5.0% of placebo-treated patients, sample sizes of at least 434 and 140 patients per group would be required to detect (power = 0.8, α-error = 0.05) drug-induced twofold and threefold increases in the background incidence, respectively. If an adverse effect is observed in 10.0% of control-group subjects, minimum sample sizes of 199 and 62 patients per group should be required for detecting twofold and threefold increases in the background incidence, respectively. The foregoing exercise on the estimation of minimum sample sizes required for achieving a given study power illustrates that clinical trials shown in Table 1 (group sizes ≤ 30) were undersized to provide a meaningful set of data on the safety of FEN.

**Figure 2:**
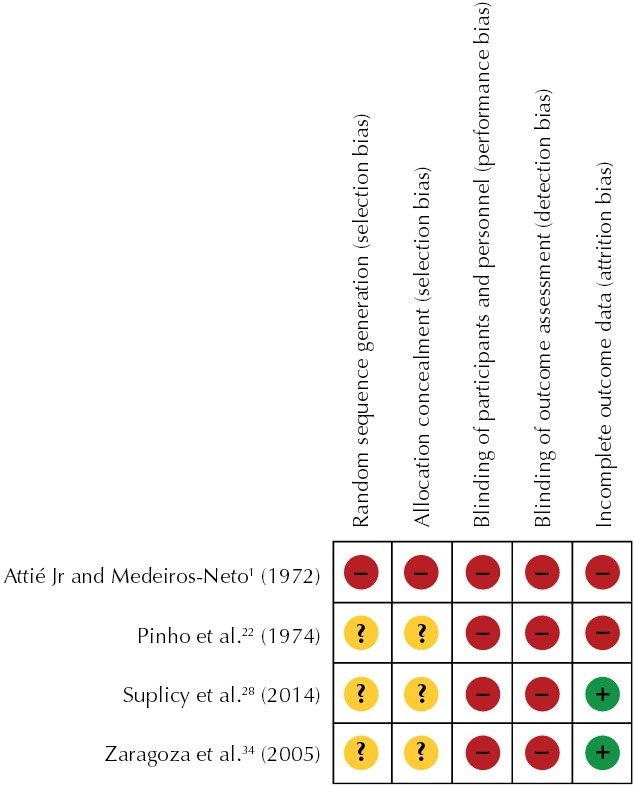
Risk of bias summary for the included studies.

Due to the previously mentioned methodological shortcomings, clinical trials of FEN showed a high risk of selection bias, performance bias, measurement bias and follow-up bias. In other words, they have low methodological quality, according to the Cochrane tool^e^ (Figure 2). The ultimate goal of pharmacological treatments of obesity is to prevent or reduce morbidity and mortality associated with excess weight and body fat, such as hypertension, cardiovascular disease, type 2 diabetes, dyslipidemia, osteoarthritis and some cancers. Therefore, weight loss is a surrogate for the “true” endpoint (reduction of morbidity). The reason why surrogate endpoints are used in clinical trial concerns reductions in the size (number of patients) or duration and cost, i.e., its feasibility in the economic scenario behind drug development process. At any rate, surrogate endpoints should be validated in long-term studies to confirm that they are in fact associated with the clinical outcome of interest. As far as antiobesity drugs are concerned, a drug that improves surrogate endpoints (i.e., promotes weight loss) may not ultimately improve more clinically relevant outcomes (i.e., “true” endpoints). The findings from the Sibutramine Cardiovascular Outcome Trial (SCOUT)^16^, a long-term clinical trial, illustrate that anorectics may have independent effects on surrogate and true endpoints. Although causing a statistically significant weight reduction, sibutramine, an anorexic drug with adrenergic properties, not only failed to reduce but also increased the risk of cardiovascular adverse effects (e.g., heart attacks) in obese patients^16^. Contrasting to the post-marketing SCOUT study, clinical studies of FEN, like most pre-marketing trials of antiobesity drugs, were not designed to evaluate efficacy considering long-term morbidity reduction. As mentioned before, all clinical studies reviewed by the authors failed to show FEN-induced improvements in secondary efficacy (surrogate) endpoints such as blood pressure and lipid parameters. Only “healthy” obese individuals were selected to participate in these controlled trials of FEN. The preselection of patients without diabetes and cardiovascular diseases precludes extrapolation of outcome data from trials on the safety and efficacy of FEN in obese patients with these comorbidities.

Red: (-) high risk of bias; Green: (+) low risk of bias; Yellow: (?) unclear risk of bias

Moreover, the present review found no clinical study supporting allegations that FEN (and the amphetamines) could be of therapeutic value for a subgroup of obese patients who failed to respond to other antiobesity drugs. The existence of such a subpopulation of obese patients who would respond better to amphetamine-like anorectics (including FEN) is at best an untested hypothesis.

In conclusion, use of FEN to treat obesity is not based on appropriately designed and soundly conducted randomized, controlled clinical trials. It is astonishing that despite the lack of good quality empirical evidence supporting its use, FEN was marketed in Latin America (particularly in Brazil and Mexico) and some European countries for over three decades. Furthermore, the human body metabolized FEN into amphetamine and many of its short-term adverse effects described in both controlled (Table 1) and uncontrolled (Table 2) clinical trials are consistent with stimulatory effects of amphetamine on the central nervous system. Amphetamine-like pharmacological properties also raise concerns regarding FEN’s abuse liability or potential of being used for nontherapeutic purposes, sporadically or even repeatedly. Abuse liability often results in psychic or physical dependence and addiction, a chronic disorder characterized by compulsive use or impaired control over drug use, and continued use despite harm and desire to stop using. At least four cases of abuse and dependence on FEN have been reported in the medical literature^6^ ^,^ ^21^ ^,^ ^27^ (Table 3).

**Table 3:** Cases of abuse and dependence on fenproporex (FEN) reported in the medical literature.

Case description	Duration of drug use	Withdrawal symptoms	Remarks	Reference
France, woman, aged 45 years, FEN prescribed for weight loss	5 years	Compulsive seeking for the drug, aggressive behavior, anxiety, insomnia, irritability.	FEN consumption stopped when the drug was no longer available in the market.	Pélissier-Alicot et al.^21^ (2006)
United States, woman, aged 29 years	4 years	Cravings, tremor, headache, and anxiety appeared when patient attempted to cut down use.	Drug (“Brazilian diet pill”) was illicitly imported from Brazil.	Cohen^6^ (2009)
United States, man (truck driver), aged 38 years	Not reported	Palpitations and insomnia when making regular use of FEN-based pills.	Drug illicitly imported from Brazil. After a positive amphetamine urine test, patient was suspended from his job, stopped using the pills and symptoms were resolved.	Cohen^6^ (2009)
United States, woman, aged 26 years	2 years	Intermittent chest pain, palpitations, headache, insomnia, fatigue and nausea while making use of FEN. Cravings and depressive symptoms appeared when patient stopped taking the pills.	Drug was illicitly imported into the United States. Urine toxicological screen detected amphetamine and benzodiazepines.	Smith and Cohen^27^ (2010)

## Footnotes

a European Agency for the Evaluation of Medicinal Products. Final opinion of the Committee for Proprietary Medicinal Products in accordance with article 12 of directive 75/319/EEC as amended. London; 1996. (CPMP/101-109/96). Available from: http://www.ema.europa.eu/docs/en_GB/document_library/Referrals_document/Anorectics_31/WC500014209.pdf

b Agência Nacional de Vigilância Sanitária. Resolução RDC nº 52, de 6 de outubro de 2011. Dispõe sobre a proibição do uso das substâncias anfepramona, femproporex e mazindol, seus sais e isômeros, bem como intermediários e medidas de controle da prescrição e dispensação de medicamentos que contenham a substância sibutramina, seus sais e isômeros, bem como intermediários e dá outras providências. *Diario Oficial Uniao*. 10 out 2011; Seção 1:55.

c Congresso Nacional. Decreto legislativo nº 273 de 2014. Susta a Resolução - RDC nº 52, de 6 de outubro de 2011, da Agência Nacional de Vigilância Sanitária - ANVISA, que dispõe sobre a proibição do uso das substâncias anfepramona, femproporex e mazindol, seus sais e isômeros, bem como intermediários e medidas de controle da prescrição e dispensação de medicamentos que contenham a substância sibutramina, seus sais e isômeros, bem como intermediários. *Diario Oficial Uniao*. 5 set 2014; Seção 1:1.

d Associação Médica Brasileira; Conselho Federal de Medicina. Projeto Diretrizes: obesidade e sobrepeso: tratamento farmacológico. Brasília (DF); 2010 [cited 2015 Sept 15]. Available from: http://www.projetodiretrizes.org.br/diretrizes10/obesidade_e_sobrepeso_tratamento_farmacologico.pdf

e Higgins JPT, Green S, editors. Cochrane handbook for systematic reviews of interventions: version 5.1.0 [updated Mar 2011]. London: The Cochrane Collaboration; 2011. Available from: http://www.cochrane-handbook.org

f Attié Jr and Medeiros-Neto informed that the prescribed posology was 2 tablets of Lipenan® (SARSA Laboratories) in the morning and one in the evening. Nonetheless, the authors omitted the amount of FEN per tablet. According to Pinho et al, each tablet of Lipenan® contained as much as 11.2 mg of the active substance (FEN).
